# Supplementary material for: Genome-wide CRISPR Screens in T Helper Cells Reveal Pervasive Crosstalk between Activation and Differentiation
Source: Cell. 2019 Feb 7;176(4):882–896.e18. doi: 10.1016/j.cell.2018.11.044 (PMC6370901; doi:10.1016/j.cell.2018.11.044)
Supplement: Data S2. Processed Data from All the Steps of the Analysis, Related to Figure 1 [file mmc2.zip › supplemental data/motif analysis/Pparg_homer/homerResults/motif11.info.html]

Motif 11

## Information for 12-TTATGACTGTAC (Motif 11)

A
C
G
T
A
G
C
T
G
T
C
A
G
A
C
T
A
C
T
G
T
G
C
A
T
A
G
C
C
G
A
T
T
A
C
G
G
C
A
T
C
T
G
A
G
T
A
C
  
Reverse Opposite:
